# Discussions at the Ohio State Dental Society

**Published:** 1880-01

**Authors:** E. G. Betty


### 14th ANNUAL MEETING,

—OF THE—

### Ohio State Dental Society,

### AT COLUMBUS, OHIO,

### December 3rd, 4th and 5th, 1879.

**Reported by E. G. BETTY, D. D. S.**

### DISCUSSIONS.—FIRST DAY.

### MORNING SESSION.

The first subject on the programme—"Pathology of the osseous structure of the teeth," was passed by default. Dr. Siddal had promised to read a paper, but did not put in an appearance. The second subject—"Artificial crowns engrafted upon the roots of natural teeth," was then taken up for discussion.

*Dr. F. A. Hunter* said he was glad to see a disposition amongst the members of the profession, to return to pivot teeth, and noted the many papers upon the subject, which have appeared in the dental periodicals within the past few months; said it was a mistake to extract the anterior teeth, especially the incisors. He saves good natural teeth which have been extracted, for the purpose of introducing them in the mouth in suitable cases. Marshall Webb's process of mounting a plate tooth on a small plate covering the end of the root, and securing it by means of a wire inserted into the root and fastened with "Hill's stopping," often proved of service. He said that Richmand's hollow gold crown, embracing the end of the root or roots, is valuable when nothing else can be done.

*Dr. A. L. Brown,* asked Dr. Hunter his method of mounting a natural crown.

*Dr. Hunter.*—Cut off the root at the neck of the tooth, enlarge the pulp canal, insert strong platinum wire into the root, plugging with gold to make water-tight, and attach the crown to the wire with cement.

*Dr. Brown* illustrated upon the black-board, his method of engrafting bicuspid teeth. He said that though it was difficult to mount these teeth, yet the roots could be used and should not be extracted. Take platinum plate number 24 of the gauge plate, and make flat pivot. Burr out the root in the form of a dove-tail, and plug the pivot in tightly with amalgam; Then fit a band or collar of No. 32 gauge platinum plate, to a plate tooth, solder the platinum to the tooth with pure gold; take impression of the end of the root, and accurately adjust the band, making it fit closely to the end of the root; then fill the dove-tail in the root, and the hollow band or collar with amalgam. This method makes a firm operation and gives a solid masticating surface. The incisor teeth may be treated pretty much in the same way, only the band is dispensed with. Dress the root below or under the free margin of the gum, cupping the end. Drill out the canal so as to receive a heavy platinum wire, which is fastened to the artificial crown; the tooth is then "backed up" with amalgam, the cupping serving to make it secure and protect the end of the root. In the use of wooden pivots, trouble results from the entrance of moisture, and consequent decay.

*Dr. D. R. Jennings* said that he had been experimenting with bicuspids for two years. He made the crown of the tooth on the Richmond plan, substituting platinum for the gold After filling the apex of the root, the canal is reamed out to receive a strong steel screw, which is adjusted very firmly. For the artificial crown, he uses this platinum plate, the band fitting snugly over the root, but not tight enough to necessitate driving it to its place. The bottom of the cap immediately over the root, is filled with oxychloride of zinc, a capping of amalgam over this, giving a solid grinding surface. In one of the first cases treated in this way, the steel screw in the root after a year's use, when taken out, was bright, while the part imbedded in the cement, though it had not rusted, was turned of a brown color. During the past two years, he had used this method in about a hundred cases. For molars, an impression is taken of the roots for fitting the band or collar. The steel screw is inserted into each of the roots, and the band neatly adjusted. The grinding surface can be made of either platinum or amalgam. It is a great mistake to use force to drive the band over the roots and down to the process

*Dr. Hunter* exhibited models of several bicuspids of which the buccal cusps were gone. For treating such, he takes an impression of the remaining part of the tooth, and the gum around it; select a plain plate cuspid tooth, back it with platinum, and grind to model; solder to the backing, a wire, for insertion into the root, and to the anterior and posterior edges; solder the ends of a platinum band so as to go under the margin of the gum; fasten the wire into the root with oxychloride of zinc and fill the tube or band with amalgam.

*Dr. J. Taft.*—Efforts being made in this direction, show an appreciation of the value of natural roots, both for the sake of contour of the face and the position of the neighboring teeth, as well as for the reception of artificial crowns. It is a serious mistake to extract roots merely for the sake of inserting a plate, while the indications are so gratifying that by the methods just detailed, they can be made to serve a useful purpose. Some take up a special method and follow it in all cases, but the better plan is to study each case and use that method which indicates the best results. One practioner uses a modification of Richmond's method to meet peculiarities of the case. Amalgam is admissible in such operations as these. Steel or iron screws may be used and the rust, if it takes place, would do no harm, at all events, but a small portion would be exposed to moisture. The progress made in the preservation of roots, is very gratifying.

*Dr. F. H. Rehwinkel* entirely agrees with Dr. Taft. Dr. Brown's process is as good as can be suggested. If gold is to be used, it may be so combined by soldering, that the gold can be exposed externally, leaving the platinum inside to receive the amalgam, as it does not readily combine with the mercury. His experience is that steel screws may be used with propriety, provided they are perfectly protected from moisture. In one case the steel screw was exposed at the end, through the grinding surface, and upon removing it, after eight or nine months, a black deposit was found around it and upon the gold. An examination of the screw revealed an opening all along it, the screw being almost entirely destroyed; the result of electrical action. In Webb's method there is danger of splitting the root in packing the pivot with gold. To overcome this, he introduces a platinum cylinder into the root securing it with amalgam. A second cylinder of platinum is made to fit within the first, so that the tooth may be withdrawn; finally the gold pivot carrying the tooth and plate, is adjusted into the second cylinder, the joint between the plate and the end of the root being protected with a layer of gutta percha. The natural roots should be saved, if possible, thus restoring, in a measure, the original use of the tooth.

*Dr. H. A. Smith* noticed an article in an English journal advising the preservation of the natural roots. He likes the Richmond method, the only objection to it being the difficulty of making a tight joint at the end of the root, and the liability of periostitis and loosening of the roots.

*Dr. J. Taft* said that this objection did not always obtain. The wasting away of the steel pivot spoken of by Dr. Rehwinkel, could be obviated by perfectly covering the steel.

*Dr. A. L. Brown* had an artificial crown in his own mouth, put on by Dr. Rehwinkel, in which the end of the screw was exposed at the grinding surface, so that galvanic action occurred, inducing periostitis; but the end was cut out and covered with gold; then the difficulty ceased.

*Dr. H. A. Smith* says there will be no periostitis, if the periosteum is not injured by stripping it from the root.

*Dr. Herriott* does not like to use anything in the front of the mouth, which does not look like the natural tooth, porcelain should be used as a face for the tooth. In the back of the mouth, the metallic crowns will do well enough. Gold screws can be used instead of steel, and thus obviate any trouble arising from galvanic action. It is important to save a root for the sake of its antagonist, which is liable to be thrown out by nature, when its use ceases.

### AFTERNOON SESSION.—FIRST DAY.

### SECOND SUBJECT RESUMED.

*Dr. Corydon Palmer* says the Richmond method is well received in New York. He has found that in the superior incisors, there are many cases in which the crown interferes with the inferior teeth. For the anterior teeth, he inserts a tube into the root, securing with cement, and fastenes the pivot with red gutta-percha. For molars, the attachment is made with a screw into each root, fits a band around them and loads it with amalgam.

*Dr. W. P. Horton*: When the crown is gone clear to the margin of the gum, and the pulp remains alive, he thinks no treatment is so good as that already described, though in case of living pulp, he would load the band with oxychloride of zinc.

Subject passed.

Third Subject: New methods of mounting artificial teeth, was then taken up by the society, in connection with the second subject.

*Dr. J. Taft* made inquiries as to who had experience in testing Holbrook's method of inserting artificial plates. As he understood it, it consists in trimming the model to relieve undue bearing on hard tissue. He also instanced a method by a Chicago dentist, in which the usual quantity of plate is much reduced.

*Dr. Buffett* stated that he had seen the model trimmed by an operator twenty-three years ago, so that Holbrook's process was not essentially new.

*Dr. A. Berry* is satisfied that the Holbrook plan is an improvement; the trimming making a better fit where one part of the mouth is very hard and another soft. The soft parts are those which need the trimming so as to bring the plate to a uniform, solid bearing.

*Dr. Herriott* prevents the rocking of plates, by relieving the pressure of the plate on a hard part of the mouth, or ridge.

*Dr. Berry*: As there is nothing new under the sun, a man who brings up a forgotten thing or operation, confers upon us, a great favor.

*Dr. F. A. Hunter* thinks Holbrook's method is not essentially a trimming of the cast.

*Dr. H. A. Smith*: The secret of the patent, lies in the preparation of lower plates. The under part is so cut away that the bearing is thrown upon the sides of the ridge, leaving a large air chamber immediately over the ridge.

*Dr. J. H. Warner*: The process is nothing more nor less than an air chamber relieving pressure in one place and putting it somewhere else. The air chamber at best, is a nuisance; it irritates and inflames the mucous membrane and shortly becomes filled up. Its use should be avoided, whenever possible. He rarely uses it, and thinks that in the lower jaw, it is inadmissible, as it reduces the bearing surface and irritates the mucous membrane where it does bear.

*Dr. J. Taft*: There is still another method used in England. Make the plate as usual, attaching the teeth to pivots soldered to the plate, and passing through the crown, securing the tooth to the pivot with sulphur. This method offers a more nearly natural imitation of the teeth as they were originally. The plate is made first; teeth ground and waxed to plate, the position of the pins or pivots being marked on the plate through the tubes in the teeth. The pins are then soldered to their places, and the teeth attached with sulphur, to the pins.

*Dr. C. R. Butler*, This method of the English is to prepare the plate, grind the teeth (Ash's) closely to the plate, and mark the places for the dowels. The teeth are attached, not with nuts, but with sulphur, as it fills up all crevices and inequalities. It makes a good presentation to the tongue, and gives an expression to the teeth that can not be secured with block teeth. As to artificial crowns, he has not tried incisors, but he has cuspids and bicuspids. In one or two cases in which the pulp was alive, he used the band. The band, he drives up tight over the root, the "telescope crown" being filled with a good cement.

*Dr. Corydon Palmer* has a tooth in his own mouth, which he introduced himself, some three years ago. It is still in good shape and doing well; merely filled the band with cement, and forced it over the end of the root. As to trimming the model for artificial plates, the under plate is the most difficult. The edges of the plate ought not to press too hard on the soft tissues. In taking an impression of the mouth, the soft parts should not be pressed away too much. To avoid injury to the soft tissues, he made a gold plate twenty years ago, making it bear only on top of the ridge, clearing the sides; its use proved satisfactory.

*Dr. Von Bonhorst*: It makes but little difference what material is used for the plate, so the adaptation is perfect. He makes the edges of rubber plates thick enough to be finished round to avoid irritation at point of contact with soft parts. On gold plates, the edges are rounded by soldering on a wire. Failures are sometimes made by making the lower plate go way down under the tongue. It is a mistake; the plate should be shallow; does not use air chamber in upper plates.

*Dr. J. Taft*: There is too little attention given to adjusting partial plates; and the gum around the natural teeth is often diseased, inflamed and sometimes hypertrophied. The margin of the gum is to be guarded, as disease of it vitiates its secretion, and the decomposition of foreign substance lodged in the spaces, produce decay of the teeth. Would like somebody to explain how these conditions can best be avoided.

*Dr. Berry*: Dr. Taft's question is important and pertinent; says that many fine operators turn their mechanical work over to their assistants, so that, in that way, it is neglected. If a partial plate is fitted closely to the necks of the natural teeth, where there is a tendency to disease, the contact will shortly develop it fully. The plate should be trimmed away, and it will relieve the whole trouble.

*Dr. Rehwinkel*: Gold should be used for partial plates as often as possible. The condition of the mucous membrane, is to be considered in selecting the material for a plate; also, the manner of its adjustment. One point to be insisted upon is, that the artificial teeth should not be worn at night. The soft parts must be rested, and have a better opportunity to eliminate their secretion, the capillaries getting time to restore the normal condition of the circulation. The plate around the natural teeth is to be freely trimmed away, relieving the gums from undue pressure, and obviating a tendency to disease.

*Dr. J. Taft* endorses all that has been said about leaving a space between the natural tooth and the plate. If the gum is pressed upon by a closely fitting plate, it not only engenders disease of the gum, but there is danger of its extending to the alveolus. Nothing is gained by fitting the plate tightly against the teeth; it ought to be so arranged that the pressure is uniformly distributed.

*Dr. T. H. Smith* says that spring plates of rubber, force the teeth away, and produce soreness of the gums ; thinks that a perfect adaptation to the gum is best.

*Dr. Herriott* mentioned the use of a metallic centre for rubber plates, keeping the palate in a better condition, by reason of better heat conducting properties ; indorses Dr. Rehwinkel in insisting upon the removal of the plate at night.

*Dr. H. A. Smith* thinks that a rubber plate, with plugs of wood adjusted to bear on natural teeth, is good. On a gold plate, tubes could be soldered to receive the wooden pegs. Any inflammation or disease arising from contact with the plate, may be treated with topical applications of astringents and mild escharotics.

*Dr. J. W. Leyder*, described a method of Dr. Peterson, of Akron, in preparing a plate in same manner as for celluloid attachment, but using instead, Fletcher's cement, as the attaching material.

*Dr. J. Taft* suggested that good roots could be used for mounting an artificial plate, by fitting them with collars, the plate having collars large enough to receive them, the crowns to be atttached to the plate in any way desirable. It stands firmly, and is better than the use of clasps. If a root has a portion of living pulp, save it alive, if possible.

*Dr. Corydon Palmer* spoke of plates but little larger than the air chamber, having arms projecting to the space containing the teeth.

*Dr. Rehwinkel* experimented with a plate, by cutting it away as far as possible, so that it still retained its place and felt comfortable. The experiment proved satisfactory.

### SECOND DAY.

### MORNING SESSION.

*Dr. A. Berry* extracted an inferior bicuspid which had a contour gold filling in it, put in forty years ago.

*Dr. G. E. Billings* related a case which he acknowledged to be beyond his control. The patient is a young man nineteen years of age. Fillings introduced some five years ago, did well. From one to two years ago, he quit treating by filling as it failed to preserve the teeth. They are white and very sensitive, and in about one-half hour after washing, the margins of the gum become coated with a white secretion, which was removed with lemon juice, though too frequent use of it, exalted the degree of sensibility.

*Dr. J. H. Warner* says that lactoposphate of lime is a good corrective for acid secretion producing sensitiveness.

*Dr. Berry* lays the blame upon a wrong system of diet, the patient should leave off stimulants and live upon brown bread and vegetables.

*Dr. Billings*: The patient is on a moderate diet but it produces no effect. The gum has receded from around the necks of the incisors, to a greater degree from the upper ones. The necks under the gum become whiter than the balance of the tooth structure; is chalky, and pits and holes appear in these white rings or soft places.

*Dr. J. H. Warner*: What remedy has the physician prescribed? If Tinct Ferri. Muriate, it is the cause.

*Dr. Buffett* remarked that none of the various secretions, saliva, perspiration or urine, were known to be either acid or alkaline.

The food is not nourishing when the patient is not in a sufficiently healthy condition for perfect digestion to take place. Local treatment is to be used in connection with systemic.

*Dr. Billings* said, that to the best of his knowledge, the white secretion is alkaline.

*Dr. H. A. Smith,* by means of diagrams on the board, illustrated a case, where an upper central incisor was knocked out of position so that it stood out and overlaped the neighboring tooth. As it could not be replaced by force, it was extracted. The anterior face of the alveolus was broken; a large V shaped piece being lost. The pulp chamber and root canal were filled, tooth replaced and the fractured alveolus treated with thymic acid, so that the parts were restored by granulation, without inflammation; the whole treatment covering a period of two weeks. Did not scrape the periosteum from the root; after extraction, dipped the tooth in a solution of carbolic acid and wrapped with bibulous paper to keep clean. The root was filled with lead.

*Dr. C. N. Harroun*: A superior left lateral incisor was abscessed at the root, extracted the tooth, scraped the root, filled and replaced. In ten days, irritation was so bad that the tooth had to be removed. Portions of the root near the neck were so far absorbed away that the gold in the root canal was exposed to view.

*Dr. D. R. Jennings* read a small paper written by Dr. Peebles, describing a case of English plate work. A couple of partial plates on rubber, secured with clasps and springs, were put in the mouth nineteen years ago, never having been removed during all that time. The soft parts were in a horrible condition of disease, great portions having sloughed away. The bony walls were badly necrosed. The whole mouth was in a horrible condition; the odor vile. The system was in a debilitated condition, and it was not long after the removal of the plates, till the woman died. After steeping in carbolic acid, an examination of the plates showed large accumulations of salivary calculus upon the lower plate under the tongue, and upon the buccal sides of the upper.

*Dr. H. L. Ambler* sent the following sketch: Mrs A. *aet* 52, has just lost the left superior temporary cuspid. For the past year it has been quite loose, and finally the root was entirely absorbed, thus leaving the crown to fall out. A filling of gutta-percha on the distal surface, remained in place. The point of the permanent cuspid could be plainly seen, and eventually it erupted as a full sized tooth. All the rest of the teeth erupted at their regular times. I saw this case eight years after the above was written, and the cuspid referred to was still in place, together with nearly a full denture above and below.

*Dr. J. Taft* exhibited a specimen, where a single tooth on a plate, had passed into the œsophagus. The case was reported in the October number of the *Register*. He said that great care should be had in securing such small plates, so that the danger of their dropping into the throat may be avoided.

*Dr. H. A. Smith* related the case of a man who had had a dozen different plates, none of which fitted his mouth, and the last heard of him was his death; a plate having lodged in his œsophagus.

*Dr. A. F. Price* said that a young man aged 21 years, consulted him for advice in regard to his mouth. The alveolus was pushed forward to such an extent that the inferior teeth strike one-quarter of an inch in front of the superior incisors.

*Dr. Berry* said that in such cases, Dr. Hawley removed a V shaped piece from each side of the maxilla and drew it back.

*Dr. T. H. Smith* exhibited a contrivance for restoring the palate process of the superior maxillary; material, rubber

Subject passed.

AFTERNOON SESSION.

The Fifth Subject being in order, was then taken up for discussion. Dr. Watt read a paper on "Salivary Calculus and its Concomitants," and it being pretty much the same in kind, it was discussed together with the fifth subject.

*Dr. J. G. Templeton* much interested in Dr. Watt's paper. The same subject was discussed not very long ago in his society, in Pennsylvania, and a preference was shown for the use of mineral acids in the treatment. Has seen a good many cases of deposits upon the teeth in malarious districts; is sorry that dentists so much neglect the proper treatment of the deposits upon the teeth, and thus stop the absorption of tissue consequent upon their presence. He relies mostly upon one remedy, sulphate of copper, which is freely applied to the gums after the tartar has been thoroughly removed; does not recognize any such condition as Rigg's disease, but takes it as he finds it, and treats accordingly The copper is not to be applied oftener than once in five or six days. In regard to fetor of the breath, he always keeps on hand, ready for use, a solution of permanganate of potash.

*Dr. J. Taft*: The subject of fetor of the breath is important and interesting, though we are much disposed to overlook it. It is important from a social aspect, in as much as the possessor constantly offends his associates. We are sometimes made sick when we get a whiff of a really bad breath. Persons are desirous to make the best impression possible upon those with whom they associate, and if the breath is fetid, they destroy an otherwise favorable impression. Persons with the worst breath, generally want to get as close to a person as possible when they have a communication to make. Dentists are often made sick by inhaling fetid breath of their patients, while working at the chair, and ill health is frequently a consequence. The dentist himself should have a perfectly sweet breath ; a bad one ought to be enough to debar him from practicing. The tobacco user is disgusting, and loads his breath with pestilential odors. When the system is unhealthy, the lungs throw off waste material, causing fetor of the breath. The skin, glands and mucous surfaces discharge debris which is filthy and gives rise to disgusting smells.

*Dr. Berry* heard that the Hindoos dislike to come in close contact with Europeans, on account of their breath and other odors arising from them. The animal food taken into the stomach, no doubt, produces the vile odor of the breath. The exhalations from the body generally are affected by the character of the food taken into the system.

*Dr. H. A. Smith* studied the subject of fetor, lately, more than formerly, and found much interest in it. He has observed that when patients are in a nervous condition of fear and diffidence, the fetor is more marked than at other times. Peculiar odors of the breath seem to be hereditary, as he knows of families all afflicted with the same odor. Nasal catarrh is a prolific cause of fetor. Most cases arise from indigestion and if a purgative is occasionally used, the difficulty is relieved.

*Dr. J. Taft*: Exhalation from the skin is also very offensive, and it is more frequently the case if some other emunctory is tardy or clogged up, the skin taking upon itself, the duty of discharging from the system, the effete matter. When the stomach is in a state of fermentation, eructation may take place, but he thinks Dr. Berry charges the stomach with more sins than it commits, as the œsophogus is not an open tube, usually being closed. When the antrum in the superior maxillary, is diseased, its discharge will taint the breath. The most frequent cause of fetor, is the result of systemic disease, and though local treatment may be used, the main reliance is to be placed upon that directed to the constitution.

*Dr. Buffett* says that what is a perfume to one person, is a fetor to another, showing that the susceptibility of the individual is the criterion from which a fetor is to be judged. In the acute form of Bright's disease, it is impossible to remain in the room with the patient by reason of the fetor emanating from the breath, as the kidneys fail to perform their duty, and other emunctories are obliged to take up their work. Albuminous food, when not thoroughly digested, is apt to give rise to the formation of sulphuretted hydrogen.

*Dr. Watt*: Some articles of food contain a disagreeable smell, but most foods give rise to bad odors by decomposition and recombinations. The breath is the worst emanation from the body. There is a sebaceous or cheesy matter collected in the tonsils, that becomes decomposed and taints the breath with a most intolerable odor. To prevent such an occurance, the collection should be removed, a wax spatula answering the purpose nicely.

*Dr. Corydon Palmer*: We ought to deal kindly with those suffering in this way. He has learned by sad experience that systemic troubles are frequently the cause; generally indigestion and constipation of the bowels.

The gums may become spongy and inflamed after the free use of animal food, or use of tobacco. If, upon experience, anything is found to produce a fetor of the breath, it should be avoided. It is important that the bowels act regularly. Should nasal catarrh be the cause, a solution of common salt in tepid water, passed through the nose, occasionally has a good effect. Sugar may be substituted for the salt, as it can be retained longer upon the abraded surfaces.

*Dr. J. Taft* said that if the mouth is constantly open when not in use, the oxygen favors decomposition of any deposits that may be present; fetor resulting. If kept closed during the night, the mouth will be in much better condition in the morning. Those who keep their teeth most clean, have the sweetest breath. In the great majority of mouths, if the effete epithelial cells be scraped from the mucous surface, the mass gives off a bad odor, as decomposition to a greater or lesser extent, has set in. The mere fact of constipation does not produce fetor; the stopping of the excretion, from the mucous membrane lining the alimentary canal, by keepiug effete matter in the blood and necessarily forcing some other emunctory to do additional work, is the true cause of the difficulty.

Subject passed.

### THIRD DAY.

### DISCUSSION.—LAST SESSION.

Sixth Subject: "Residual alveolar Abscess: Cause and Treatment" being in order was opened for discussion.

*Dr. J. H. Warner*: A Residual alveolar abscess is formed ot the residuum from an inflammation. As alcohol and the acids coagulate albumen and the pus corpuscles, the injection of carbolic acid is contra-indicated, because the coagula it produces remains in the socket as an exciting cause for a new abscess or a continuation of the old, when the circumstances are presented, which bring on an active condition of disease.

*Dr. Buffett*: As near as I can understand it, a residual alveolar abscess, is albumen coagulated by an acid. We may have an acute or chronic form of abscess. The congestion in the acute form may be overcome by local or general depletion; the acute may be treated with escharotics and astringents, producing a condition more like the chronic. If foreign matter is present at the seat of disease, it either becomes encysted and at some future time acts as an irritant, or nature may throw it off. The seat of disease should be cleansed by thoroughly washing out, and nothing that coagulates the albumen in a chronic abscess should be used as treatment. A chronic abscess is the result of systemic conditions and should be met accordingly. An injury to a part may result in the formation of pus in six hours. In a debilitated state of the system an inflammation might go on for several years with the formation of pus.

*Dr. Templeton*: In some of these cases there seems to be necrosis and Elixir Vitriol is the best remedy he has found for its treatment; Sulphate of Copper he has also found to be useful. To cure an abscess, the pyogenic membrane is to be destroyed, as it is the membrane which secrets the pus. If there is no fistula, it is best to make an opening to the seat of the disease and treat directly. He uses either carbolic acid or aromatic sulphuric acid.

*Dr. J. H. Warner*, how do you get rid of the coagulated residuum? *Voice*: Produce an abortion.

*D. J. B. Beauman* has had more satisfactory information and experience during the last two years than ever before in treating this class of cases. Always heretofore been afraid to extract and replant. In the last year, fifty cases treated in this way with universal success. The time will come when dentists generally shall adopt this method. The idea of injecting acids, alkalies, etc., is not the proper one as it is unendurable to the patient. Extract the tooth, remove the abscess from the root and replace the tooth in the socket. The abscess results from death of the pulp and should it be situated at the bifurcation it cannot be reached by injections through the roots. After extracting the tooth and scraping the root or roots, the cavity of decay is cleaned out, also root canal; the foramen at apex is filled with a gold screw, the cavity of decay plugged and the tooth replaced in the socket.

This course of treatment is much the most thorough and more certain to result in a cure. After the tooth is prepared, wash out the socket with solution of common salt in tepid water to get rid of the clot. If any pain supervenes the replacement of the tooth, morphia may be administered to produce sleep at night.

*Dr. Watt*: Dentists overlook some conditions. The tooth receives blood from two sources. If the periosteum is healthy, it will keep up the nutrition of the tooth.

The vessels of the periosteum enlarge until they can supply the tooth and thus prevent it from becoming a foreign body. Such a tooth will be found more sensitive than dead teeth usually. If cold is taken why does that periosteum become inflamed? Not because it is less vital than any other, but because it is carrying more blood than it would in a normal condition of the tooth.

Subject passed.

Seventh Subject: Irregularities of the Teeth: was not discussed, Dr. G. W. Keely treating the theme at length in a didactic manner, illustrating his remarks with models and enlarged oil-color diagrams of the cases he has had under treatment.

